# Association of Cost-Driven Residential Moves With Health-Related Outcomes Among California Renters

**DOI:** 10.1001/jamanetworkopen.2023.2990

**Published:** 2023-03-14

**Authors:** Katherine L. Chen, Lauren E. Wisk, Teryl K. Nuckols, Paul M. Ong, Ninez A. Ponce, Joann G. Elmore, Kristen R. Choi, Claudia Nau, Frederick J. Zimmerman

**Affiliations:** 1 Division of General Internal Medicine and Health Services Research, David Geffen School of Medicine, University of California, Los Angeles; 2 Department of Health Policy and Management, Fielding School of Public Health, University of California, Los Angeles; 3 Division of General Internal Medicine, Cedars-Sinai Medical Center, Los Angeles, California; 4 Department of Urban Planning, Luskin School of Public Affairs, University of California, Los Angeles; 5 Center for Health Policy Research, University of California, Los Angeles; 6 School of Nursing, University of California, Los Angeles; 7 Department of Research and Evaluation, Kaiser Permanente Southern California, Pasadena

## Abstract

**Importance:**

Unaffordable housing is associated with adverse health-related outcomes, but little is known about the associations between moving due to unaffordable housing and health-related outcomes.

**Objective:**

To characterize the association of recent cost-driven residential moves with health-related outcomes.

**Design, Setting, and Participants:**

This cross-sectional study involved a weighted multivariable regression analysis of California Health Interview Survey data from January 1, 2011, to December 31, 2017. A population-based sample of 52 646 adult renters and other nonhomeowners in California were included. Data were analyzed from March 2, 2021, to January 6, 2023.

**Exposure:**

Cost-driven moves in the past 3 years relative to no move and to non–cost-driven moves.

**Main Outcomes and Measures:**

Five outcomes were assessed: psychological distress (low, moderate, or severe, as categorized by the 6-item Kessler Psychological Distress Scale), emergency department [ED] visits in the past year (any vs none), preventive care visits in the past year (any vs none), general health (poor or fair vs good, very good, or excellent), and walking for leisure in the past 7 days (in minutes).

**Results:**

Among 52 646 adult renters and other nonhomeowners, 50.3% were female, 85.2% were younger than 60 years, 45.3% were Hispanic, and 55.1% had income lower than 200% of the federal poverty level. Overall, 8.9% of renters reported making a recent cost-driven move, with higher prevalence among Hispanic (9.9%) and non-Hispanic Black (11.3%) renters compared with non-Hispanic White renters (7.2%). In multivariable models, compared with not moving, cost-driven moving was associated with a 4.2 (95% CI, 2.6-5.7) percentage point higher probability of experiencing moderate psychological distress; a 3.2 (95% CI, 1.9-4.5) percentage point higher probability of experiencing severe psychological distress; a 2.5 (95% CI, 0-4.9) percentage point higher probability of ED visits; a 5.1 (95% CI, 1.6-8.6) percentage point lower probability of having preventive care visits; a 3.7 (95% CI, 1.2-6.2) percentage point lower probability of having good, very good, or excellent general health; and 16.8 (95% CI, 6.9-26.6) fewer minutes of walking for leisure. General health, psychological distress, and walking for leisure were also worse with cost-driven moves relative to non–cost-driven moves, with a 3.2 (95% CI, 1.7-4.7) percentage point higher probability of experiencing moderate psychological distress; a 2.5 (95% CI, 1.2-3.9) percentage point higher probability of experiencing severe psychological distress; a 4.6 (95% CI, 2.1-7.2) percentage point lower probability of having good, very good, or excellent general health; and 13.0 (95% CI, 4.0-21.9) fewer minutes of walking for leisure. However, the incidence of preventive care and ED visits did not differ between those who made cost-driven vs non–cost-driven moves.

**Conclusions and Relevance:**

In this study, cost-driven moves were associated with adverse health-related outcomes relative to not moving and to non–cost-driven moves. These findings suggest that policies to improve housing affordability, prevent displacement, and increase access to health care for groups vulnerable to cost-driven moves may have the potential to improve population health equity, especially during the current national housing affordability crisis.

## Key Points

**Question:**

How and to what extent are residential moves due to unaffordable rent associated with health-related outcomes?

**Findings:**

In this cross-sectional study of 52 646 adult renters and other nonhomeowners in California between 2011 and 2017, 9% of participants reported making a recent cost-driven move. Relative to not moving, cost-driven moving was associated with significantly worse general health and psychological distress, fewer preventive care visits, more emergency department visits, and less walking for leisure.

**Meaning:**

These findings suggest that policies to enhance housing affordability, prevent displacement, and improve access to health care and public benefits could have important positive implications for health, particularly amid the current national housing affordability crisis.

## Teaser

This cross-sectional study uses data from the California Health Interview Survey to assess how and to what extent cost-driven residential moves are associated with health-related outcomes among adult renters and other nonhomeowners.

## Introduction

More than one-half of California renters face burdensome housing costs that may displace them from their homes.^1^ Politicians and tenant advocates have declared a housing affordability crisis^2^ that is increasingly recognized as a danger to public health.^3,4^ As affordable housing shortages spread throughout the country,^5^ population health insights from California’s housing market have implications for the health of the nation.

Cost-driven displacement is a potential consequence of unaffordable housing that encompasses both forced residential moves (eg, evictions) and reactive residential moves, defined as unforced but unwanted moves in response to escalating rents or income instability.^6,7^ Forced cost-driven moves, the majority of which involve evictions due to rental arrears,^8,9,10,11^ have been associated with numerous adverse health-related outcomes, including mental illness,^12,13^ emergency department (ED) visits,^9,14^ HIV sexual risk outcomes,^15^ deaths of despair (ie, deaths from suicide or from drug or alcohol poisoning),^16,17^ and all-cause mortality.^18^

Although reactive cost-driven moves are more than twice as prevalent as forced moves,^6,19^ few studies have considered health-related outcomes associated with them. Cost-burdened renters seeking to avoid eviction for late payment, nonpayment, or underpayment of rent may face a difficult choice between reducing other important expenses, taking on additional employment, or leaving their homes for less expensive alternatives. Such decisions may be associated with previous health-related priorities and/or subsequent health-related outcomes. The research that exists to date has found that moving in response to unaffordable housing costs is associated with increased risks of poor mental health,^20,21^ poor sleep,^22^ and unmet medical care needs.^23^ How these moves are associated with other health-related outcomes remains unclear. Notably, severe housing cost burden and displacement risk are concentrated among renters with low income and renters from racial and ethnic minority groups.^24,25,26^ Understanding the full extent of the associations between cost-driven displacement and health-related outcomes may thus provide insight on potential pathways underlying health disparities.^25,27^

We sought to describe the association between cost-driven moves and health-related outcomes among renters and other nonhomeowners who are most vulnerable to economic volatility and displacement.^26,28^ To expand beyond the literature on forced moves, we defined a cost-driven move as any move motivated by unaffordable housing costs, potentially including both reactive moves and evictions due to rental arrears. This measure is not dependent on whether a participant explicitly self-identifies as forcibly displaced, thereby reducing the intentionality bias that is introduced when researchers try to determine whether moves are volitional.^6^ To understand how the context of moving shapes these associations, we compared the health-related outcomes of people who made recent cost-driven moves both with those of people who did not recently move and with those of people who made recent non–cost-driven moves.

## Methods

### Data Sources

We performed a cross-sectional analysis of data from the California Health Interview Survey (CHIS), the country’s largest state population health survey. We focused on January 1, 2011, to December 31, 2017, surveys because they contained questions relevant to cost-driven moves and coincided with the peak of California’s pre–COVID-19 era housing affordability crisis.^29,30^ Data were analyzed from March 2, 2021, to January 6, 2023. Verbal informed consent from CHIS participants was obtained via telephone. Among screened households, adult cooperation rates were 42% to 47%.^31^ Missing responses were imputed by CHIS using a combination of logical and hot-deck imputation (reported by CHIS as <1% to 2% for most variables).^31^ We merged Census tract–level neighborhood characteristics^32,33,34,35,36^ with CHIS data for descriptive analyses only (details are available in eTable 1 in Supplement 1). This study was approved by the institutional review board of the University of California, Los Angeles. The study followed the Strengthening the Reporting of Observational Studies in Epidemiology (STROBE) reporting guideline for cross-sectional studies.

### Sample

We included 52 646 CHIS participants aged 18 years and older who rented their homes or reported another nonhomeownership arrangement. We excluded participants for whom the survey was completed by proxy to ensure accuracy of responses.

### Outcomes

We analyzed 5 health-related outcomes: general health (poor or fair vs good, very good, or excellent), psychological distress (categorized by the 6-item Kessler Psychological Distress Scale; score range, 0-24, with 0-4 indicating low distress, 5-12 indicating moderate distress, and 13-24 indicating severe distress^37,38^), preventive care visits (any vs none in the past year), ED visits (any vs none in the past year), and walking for leisure (minutes walked in the past week, estimated by multiplying the number of leisure-time walking episodes by the mean episode duration^39,40^). We restricted preventive care visit analyses to the years 2013 to 2017 because the relevant survey item was not assessed from 2011 to 2012. Models that assessed walking for leisure were restricted to adults self-identifying as physically able to walk.

### Primary Exposure

The primary exposure was a 3-level categorical variable for residential moving history in the past 3 years, constructed from CHIS questions asking, “About how long have you lived at your current address?” and “The last time you moved, what was your main reason for moving?” Options for the latter are summarized in eTable 2 in Supplement 1. Participants who moved because they “couldn’t afford mortgage/rent” were considered to have made cost-driven moves.^20,23^ As comparisons, people who moved for other reasons were classified as having made non–cost-driven moves, while those whose most recent move was more than 3 years ago were classified as having made no move.

### Covariates

All multivariable models were adjusted for self-reported individual characteristics, including sex (female or male), race and ethnicity (Hispanic, non-Hispanic Asian, non-Hispanic Black, non-Hispanic White, or other non-Hispanic race [including American Indian or Alaska Native, Native Hawaiian or other Pacific Islander, ≥2 races, and self-reported other race]), family composition (married nonparent, married parent, single nonparent, or single parent), age and square of age (years), employment status (employed full time, employed part time, unemployed and seeking work, or unemployed and not seeking work), educational attainment (less than high school diploma; high school diploma; some college, associate’s degree, or vocational school; college degree; or graduate school), income (measured as log of total household income as percentage of federal poverty level), citizenship (US-born citizen, naturalized citizen, noncitizen with green card, or noncitizen without green card), limited English language proficiency (yes or no), housing type (rental or other nonhomeownership arrangement), and urbanicity (urban or rural) determined by zip code (full covariate definitions are provided in eTable 3 in Supplement 1). Race and ethnicity were included to examine differences in the prevalence of cost-driven moves and to account for the role of racist social and economic policies in shaping mutually reinforcing risk of exposure to housing and health opportunities.^41^ Models of preventive care and ED visits also controlled for health insurance type (employment-based, Medicaid alone, Medicaid and Medicare, Medicare and other insurance, Medicare or other public insurance alone, privately purchased, or not insured).

### Statistical Analysis

All analyses included survey weights via the Svy suite in Stata software, version 16 (StataCorp LLC). We calculated weighted frequencies for the total sample and subgroups by move history, using χ^2^ tests to assess bivariate differences. The statistical significance threshold was 2-tailed *P* < .05.

To assess the associations of cost-driven moves with health-related outcomes relative to no move (primary analyses), we regressed move history on each of the 5 outcomes. We modeled general health, preventive care visits, and ED visits using logistic regression analysis, and we modeled psychological distress using a partial proportional odds model.^42^ We evaluated walking for leisure with a 2-part model, including a logistic model for the odds of having walked at least 10 minutes in the past 7 days (based on CHIS item wording and consistent with previous research^39,40^) and a generalized linear model with log link for minutes walked, conditional on having walked at least 10 minutes.^43,44^ For all models, we reported average marginal effects describing the expected difference in the outcome associated with a cost-driven move vs no move, adjusting for covariates.

To understand whether differences observed in the primary analyses were associated with moving in general or with something unique to cost-driven moving, we repeated primary analyses using non–cost-driven moves as the comparator. To account for potential ambiguity in the survey question about reasons for moving, we conducted sensitivity analyses to reevaluate moving history as housing-related moves (including cost-driven moves plus moves due to “changes in renting/lease” and “other housing related” reasons [CHIS response choices]) vs non–housing-related moves and no move. Next, we assessed alternate specifications for each outcome, including an ordinal measure of general health, a continuous measure of psychological distress, preventive care visit in the past 2 years (given uncertainty around the ideal frequency of such visits^45^), number of ED visits, and an indicator of having regularly walked for leisure.^46^

## Results

### Study Participants

Among 52 646 adults, 90.9% were renters and 9.1% were other nonhomeowners. Overall, 50.3% of participants were female, 49.7% were male, 85.2% were younger than 60 years, and 55.1% reported income lower than 200% of the federal poverty level (Table). With regard to race and ethnicity, 45.3% of participants were Hispanic, 13.2% were non-Hispanic Asian, 7.7% were non-Hispanic Black, 30.9% were non-Hispanic White, and 3.0% were of other non-Hispanic races (including American Indian or Alaska Native, Native Hawaiian or other Pacific Islander, ≥2 races, and self-reported other race). In total, 8.9% of participants reported making a cost-driven move in the past 3 years, with higher prevalence among Hispanic (9.9%) and non-Hispanic Black (11.3%) renters compared with non-Hispanic White renters (7.2%). Overall, 15.4% of all recent moves were due to cost (eTable 2 in Supplement 1).

**Table.** Characteristics of Renters and Other Nonhomeowners in the California Health Interview Survey, 2011-2017^a^

Characteristic	Participants, unweighted No. (weighted %)				*P* value
	Total (N = 52 646)	Residential moving history (past 3 y)			
		Cost-driven move (n = 3747)	No move (n = 27 558)	Non–cost-driven move (n = 21 341)	
**Participant characteristics**					
Total participants, weighted %	100	8.9	42.4	48.7	NA
Housing type					
Rental	47 160 (90.9)	3466 (92.8)	24 076 (87.5)	19 618 (93.4)	<.001
Other nonhomeownership arrangement	5486 (9.1)	281 (7.3)	3482 (12.5)	1723 (6.6)	
Sex					
Female	30 158 (50.3)	2123 (50.3)	16 160 (51.1)	11 875 (49.6)	.23
Male	22 488 (49.7)	1624 (49.7)	11 398 (48.9)	9466 (50.4)	
Age group, y					
18-24	6264 (16.5)	530 (17.1)	2221 (13.1)	3513 (19.4)	<.001
25-34	8610 (27.9)	737 (28.6)	2732 (19.7)	5141 (34.9)	
35-49	11 946 (28.4)	997 (30.7)	5878 (29.9)	5071 (26.7)	
50-59	9255 (12.4)	672 (13.3)	5529 (15.8)	3054 (9.2)	
60-69	8025 (8.1)	516 (7.2)	5185 (11.3)	2324 (5.6)	
70-79	5050 (4.3)	202 (2.2)	3555 (6.7)	1293 (2.7)	
≥80	3496 (2.4)	93 (0.9)	2458 (3.6)	945 (1.5)	
Race and ethnicity					
Hispanic	18 482 (45.3)	1551 (50.5)	9873 (49.9)	7058 (40.2)	<.001
Non-Hispanic Asian	5435 (13.2)	307 (11.8)	3124 (12.2)	2004 (14.2)	
Non-Hispanic Black	3902 (7.7)	322 (9.8)	2020 (7.3)	1560 (7.8)	
Non-Hispanic White	22 649 (30.9)	1424 (25.0)	11 502 (27.9)	9723 (34.5)	
Other^b^	2178 (3.0)	143 (2.9)	1039 (2.7)	996 (3.3)	
Family composition					
Married nonparent	7899 (13.7)	489 (11.1)	4660 (15.9)	2750 (12.2)	<.001
Married parent	8718 (22.2)	738 (23.4)	4008 (21.7)	3972 (22.5)	
Single nonparent	31 262 (54.1)	2065 (52.2)	16 825 (53.3)	12 372 (55.1)	
Single parent	4767 (10.0)	455 (13.2)	2065 (9.1)	2247 (10.2)	
Employment					
Employed full time	23 153 (56.1)	1774 (55.3)	10 716 (52.0)	10 663 (59.7)	<.001
Employed part time	4845 (9.9)	366 (10.1)	2508 (10.5)	1971 (9.3)	
Unemployed and seeking work	3710 (8.5)	398 (11.6)	1682 (7.5)	1630 (8.7)	
Unemployed and not seeking work	20 938 (25.6)	1209 (23.0)	12 652 (29.9)	7077 (22.2)	
Educational attainment					
Less than high school diploma	10 144 (22.5)	781 (25.5)	6013 (27.2)	3350 (18.0)	<.001
High school diploma	14 296 (23.9)	1114 (25.7)	7422 (24.3)	5760 (23.2)	
Some college, associate’s degree, or vocational school	13 917 (25.7)	992 (26.4)	7239 (25.1)	5686 (26.0)	
College degree	9263 (19.5)	578 (16.7)	4447 (16.4)	4238 (22.7)	
Graduate school	5026 (8.4)	282 (5.7)	2437 (7.1)	2307 (10.1)	
Income, % of federal poverty level					
0-99	15 558 (28.9)	1292 (34.4)	8357 (29.9)	5909 (27.1)	<.001
100-199	14 582 (26.2)	1104 (28.7)	8157 (28.4)	5321 (23.9)	
200-299	7624 (15.1)	544 (15.3)	3934 (15.1)	3146 (15.0)	
≥300	14 882 (29.8)	807 (21.6)	7110 (26.7)	6965 (34.1)	
Limited English language proficiency	10 794 (23.0)	810 (25.5)	6577 (28.2)	3407 (18.0)	<.001
Health insurance type					
Employment-based	13 982 (32.8)	891 (25.9)	6349 (28.5)	6742 (37.7)	<.001
Medicaid only	12 703 (26.7)	1119 (31.6)	6178 (27.3)	5406 (25.3)	
Medicaid plus Medicare	7195 (6.6)	359 (5.3)	5063 (9.7)	1773 (4.2)	
Medicare plus other insurance	5337 (4.2)	220 (1.9)	3434 (6.0)	1683 (3.0)	
Medicare or other public insurance only	2881 (3.9)	184 (3.2)	1604 (4.2)	1093 (3.7)	
Private	2567 (5.4)	191 (5.3)	1171 (4.6)	1205 (6.1)	
No insurance	7981 (20.5)	783 (27.0)	3759 (19.7)	3439 (19.9)	
Immigration and citizenship					
US-born citizen	34 656 (58.9)	2386 (54.4)	17 369 (54.7)	14 901 (63.3)	<.001
Naturalized citizen	8253 (14.9)	532 (14.9)	5355 (18.8)	2366 (11.5)	
Noncitizen with green card	5159 (12.2)	400 (13.5)	2738 (12.6)	2021 (11.5)	
Noncitizen without green card	4578 (14.1)	429 (17.3)	2096 (13.9)	2053 (13.8)	
Urbanicity					
Urban	44 859 (92.3)	3186 (92.8)	23 631 (92.4)	18 042 (92.1)	.57
Rural	7787 (7.7)	561 (7.2)	3927 (7.6)	3299 (7.9)	
**Census tract characteristics^c^**					
Social Vulnerability Index, California state quartile					
1 (least vulnerable)	8419 (16.1)	497 (12.8)	4206 (14.5)	3716 (18.1)	<.001
2	12 467 (22.2)	829 (19.7)	6295 (20.2)	5343 (24.4)	
3	14 493 (28.0)	1107 (30.6)	7560 (28.5)	5826 (27.0)	
4 (most vulnerable)	17 083 (33.8)	1306 (36.9)	9418 (36.9)	6359 (30.5)	
Incarceration rate, California state quartile					
1 (lowest rate)	10 599 (19.1)	645 (15.4)	5516 (18.1)	4438 (20.7)	<.001
2	12 504 (23.3)	861 (22.3)	6489 (22.8)	5154 (23.8)	
3	13 909 (26.7)	1048 (28.2)	7297 (26.8)	5564 (26.4)	
4 (highest rate)	15 450 (30.9)	1185 (34.1)	8177 (32.3)	6088 (29.2)	
Walkability Index, California state quartile					
1 (most walkable)	15 167 (32.4)	969 (30.8)	8467 (33.5)	5731 (31.6)	.003
2	12 100 (26.3)	850 (26.4)	6491 (27.0)	4759 (25.7)	
3	13 166 (23.8)	960 (24.2)	6643 (23.3)	5563 (24.2)	
4 (least walkable)	12 029 (17.5)	960 (18.6)	5878 (16.2)	5191 (18.5)	
Park access, California state quartile					
1 (most access)	11 977 (26.4)	767 (25.9)	6652 (27.0)	4558 (26.1)	.70
2	13 368 (25.7)	1003 (26.1)	6994 (26.0)	5371 (25.5)	
3	13 341 (24.6)	937 (25.4)	6803 (24.3)	5601 (24.7)	
4 (least access)	13 776 (23.3)	1032 (22.5)	7030 (22.8)	5714 (23.8)	
Housing and Transportation Affordability Index, California state quartile					
1 (most affordable)	16 794 (36.2)	1180 (37.0)	9415 (38.7)	6199 (34.0)	<.001
2	13 781 (26.9)	968 (25.4)	7204 (27.2)	5609 (26.9)	
3	12 658 (22.3)	965 (24.0)	6352 (21.3)	5341 (22.9)	
4 (least affordable)	9229 (14.5)	626 (13.7)	4508 (12.8)	4095 (16.2)	

Abbreviation: NA, not applicable.

^a^ *P* values are from 2-tailed χ^2^ tests of differences in variable frequencies across residential moving status groups. Column percentages may not add up to 100 due to rounding.

^b^ Includes non-Hispanic American Indian or Alaska Native participants, non-Hispanic Native Hawaiian or other Pacific Islander participants, non-Hispanic participants describing their race as other, and non-Hispanic participants selecting 2 or more races.

^c^ Reported for the 52 462 participants (99.7% of total sample) living in Census tracts for which all measured Census tract characteristics were nonmissing.

Participants reporting cost-driven moves were disproportionately from socioeconomically marginalized groups compared with those reporting no move or non–cost-driven moves (Table). For example, among those who made a cost-driven move, 50.5% were Hispanic, 9.8% were non-Hispanic Black, 13.2% were single parents, 11.6% were unemployed and seeking work, 63.1% had low income (ie, earned less than 200% of the federal poverty level), 27.0% were uninsured, 17.3% were noncitizens without green cards, and 67.5% were living in Census tracts with higher social vulnerability (ie, higher than the median on the Social Vulnerability Index [California state quartiles 3 and 4]). In comparison, among those who did not move, 49.9% were Hispanic, 7.3% were non-Hispanic Black, 9.1% were single parents, 7.5% were unemployed and seeking work, 58.3% had low income, 19.7% were uninsured, 13.9% were noncitizens without green cards, and 65.4% were living in Census tracts with higher social vulnerability. Among those who made a non–cost-driven move, 40.2% were Hispanic, 7.8% were non-Hispanic Black, 10.2% were single parents, 8.7% were unemployed and seeking work, 51.0% had low income, 19.9% were uninsured, 13.8% were noncitizens without green cards, and 57.5% were living in Census tracts with higher social vulnerability. Incarceration rates were also higher among those who made cost-driven moves (62.3% lived in Census tracts with incarceration rates higher than the median [California state quartiles 3 and 4]) compared with those who did not move (59.1% lived in Census tracts with incarceration rates higher than the median) and those who made non–cost-driven moves (55.6% lived in Census tracts with incarceration rates higher than the median).

### Primary Analyses: Comparison With Not Moving

**Figure 1.**
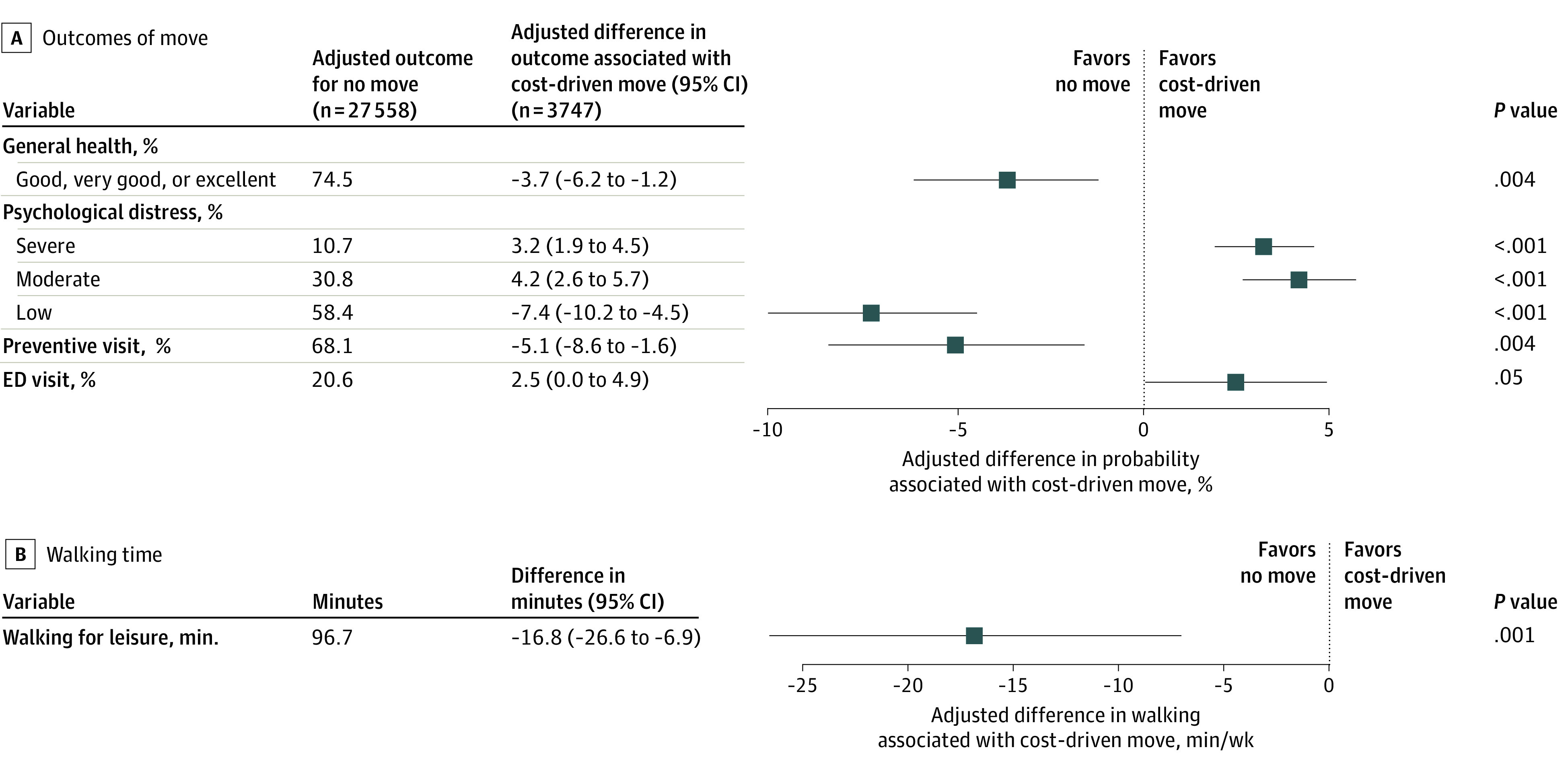
Adjusted Associations Between Cost-Driven Residential Moves and Health-Related Outcomes Relative to No Residential Move Unweighted sample sizes are shown; all other values represent weighted estimates. Average marginal effects are shown in the forest plot, with 95% CIs indicated by whiskers. All models were adjusted for sex, race and ethnicity, family composition, age, age squared, employment status, educational attainment, income, housing type, urbanicity, limited English language proficiency, citizenship, and survey year. Preventive care visit and ED visit models were also adjusted for health insurance type. ED indicates emergency department.

Cost-driven moving was associated with adverse health-related outcomes in all 5 categories compared with not moving. Relative to those who did not move and adjusted for covariates, participants reporting cost-driven moves were less likely to report having good, very good, or excellent general health (adjusted difference, −3.7 [95% CI, −6.2 to −1.2] percentage points); more likely to report experiencing moderate psychological distress (adjusted difference, 4.2 [95% CI, 2.6 to 5.7] percentage points); more likely to report experiencing severe psychological distress (adjusted difference, 3.2 [95% CI, 1.9 to 4.5] percentage points); less likely to have a preventive care visit (adjusted difference, −5.1 [95% CI, −8.6 to −1.6] percentage points); and more likely to visit the ED (adjusted difference, 2.5 [95% CI, 0 to 4.9] percentage points) (Figure 1). Those who made cost-driven moves also spent 16.8 (95% CI, 6.9-26.6) fewer minutes per week walking for leisure.

### Comparison With Non–Cost-Driven Moving

**Figure 2.**
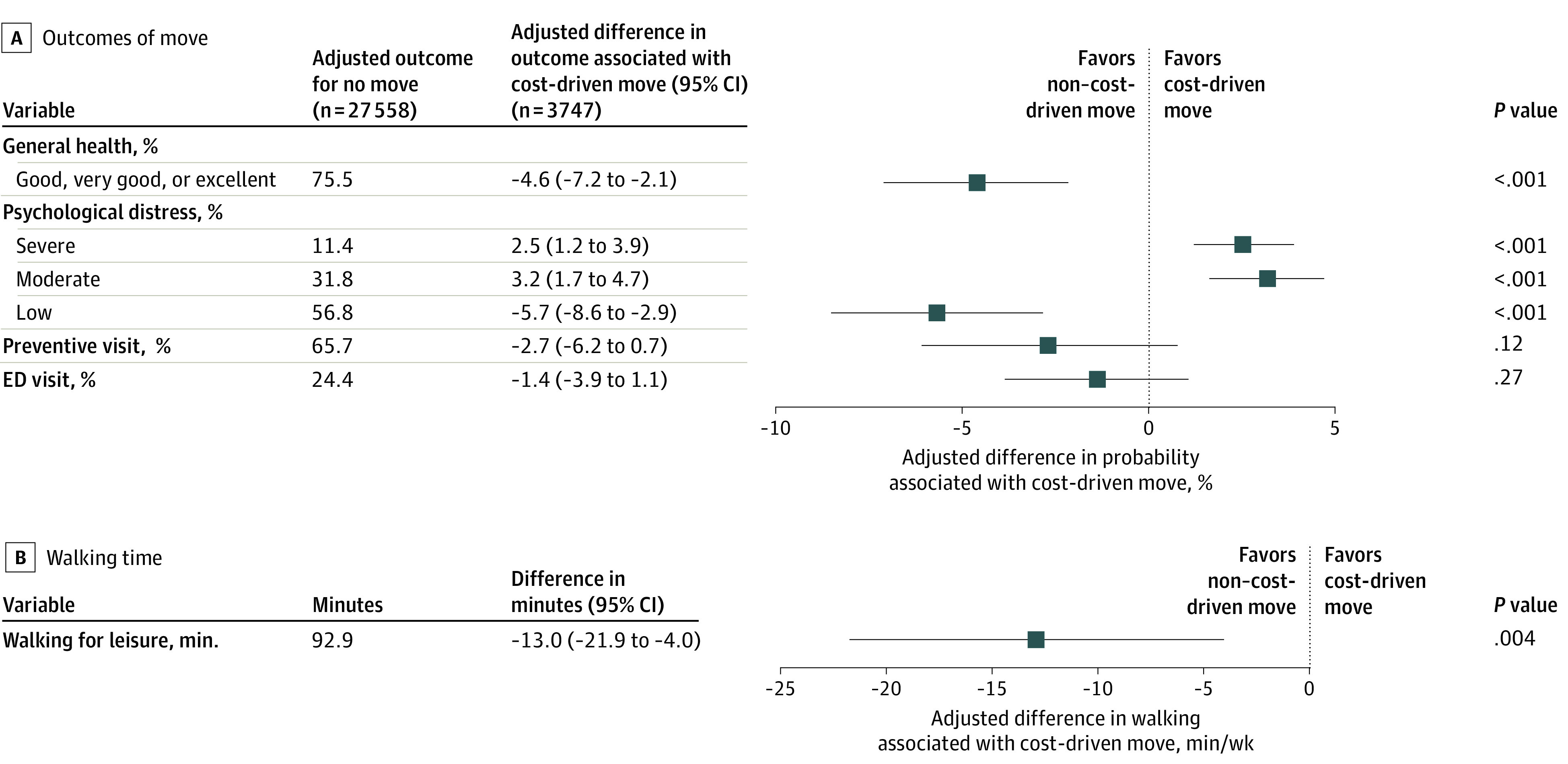
Adjusted Associations Between Cost-Driven Residential Moves and Health-Related Outcomes Relative to Non–Cost-Driven Residential Moves Unweighted sample sizes are shown; all other values represent weighted estimates. Adjusted outcomes for no move represent the reference values. Average marginal effects are shown in the forest plot, with 95% CIs indicated by whiskers. All models were adjusted for sex, race and ethnicity, family composition, age, age squared, employment status, educational attainment, income, housing type, urbanicity, limited English language proficiency, citizenship, and survey year. Preventive care visit and ED visit models were also adjusted for health insurance type. ED indicates emergency department.

General health, distress, and walking for leisure were also significantly worse among those who made cost-driven moves relative to those who made non–cost-driven moves (Figure 2). Participants who made cost-driven moves were less likely to report having good, very good, or excellent general health (adjusted difference, −4.6 [95% CI, −2.1 to −7.2] percentage points); more likely to report experiencing moderate psychological distress (adjusted difference, 3.2 [95% CI, 1.7 to 4.7] percentage points); and more likely to report experiencing severe psychological distress (adjusted difference, 2.5 [95% CI, 1.2 to 3.9] percentage points) compared with people who made non–cost-driven moves. Those who made cost-driven moves also walked 13.0 (95% CI, 4.0-21.9) fewer minutes per week for leisure. The likelihood of having preventive care visits (adjusted difference, −2.7 [95% CI, −6.2 to 0.7] percentage points) and ED visits (adjusted difference, −1.4 [95% CI, −3.9 to 1.1] percentage points) did not differ between those who made cost-driven vs non–cost-driven moves.

### Sensitivity Analyses

Patterns of associations with health-related outcomes were consistent when the primary exposure was formulated more broadly as housing-related moves (eTable 4 in Supplement 1). Slightly attenuated estimates in these models suggested that this broad category likely captured a more heterogeneous group of movers. For alternate outcome specifications, we observed consistent associations between cost-driven moves and adverse health-related outcomes, but some models had greater imprecision (eTable 5 in Supplement 1).

## Discussion

In this cross-sectional study of population-based survey data from 2011 to 2017, 15.4% of recent moves by California renters and other nonhomeowners were due to unaffordable housing costs. These cost-driven moves were most prevalent among socioeconomically marginalized groups and were associated with many adverse health-related outcomes, including worse general health, greater psychological distress, lower likelihood of having a preventive care visit, higher probability of having an ED visit, and less time spent walking for leisure compared with no move. Our results emphasized that cost-driven moves, even beyond formal evictions, are a prevalent experience that is consistently associated with poor health among renters and other nonhomeowners.

Stress associated with cost-driven moves may be a major explanation for our findings. The association of cost-driven moves with both moderate and severe psychological distress adds to the results of previous studies,^20,21^ which found that these cost-driven moves were associated with increased risks of anxiety attacks^20^ and mental discomfort.^21^ Moving for any reason typically costs money and requires adjustments in daily routines, resulting in at least short-term stress.^47,48^ However, our secondary analyses suggested that the association between cost-driven moves and adverse health-related outcomes extended beyond the stress of moving itself, given that negative associations persisted when we compared cost-driven vs non–cost-driven moves. These differences suggest that the turmoil of cost-driven moves could produce a distinctly enduring form of postmove stress that has negative implications for health. Previous research^7^ has found that when families with lower income move in response to sudden and unpredictable events, like a rent increase or loss of income, they often make subsequent housing decisions based on prioritizing immediate survival needs. Such decisions often come at the expense of longer-term advantages, such as neighborhood or school quality, which could limit health opportunities for years to come and increase their likelihood of moving again soon.^7^

Our finding that cost-driven moves were associated with significantly worse general health expands on the findings of 2 previous studies,^20,21^ which also found that those who made cost-driven moves had worse general health; however, the findings in those studies^20,21^ were not statistically significant, but the studies may have been underpowered. The negative association between cost-driven moves and general health may be explained not only by the physiological toll of stress on general health^49^ but also by the fact that stress often results in to decreased participation in health-promoting behaviors and increased uptake of unhealthy coping mechanisms,^50^ especially among people with fewer resources to support adaptation and resilience. For instance, stress may be associated with reduced participation in physical activity,^51^ which would also explain the negative association between cost-driven moves and walking for leisure observed in the current study. In addition, cost-driven moves may necessitate compromises (eg, working more hours, reducing certain expenses, or moving to lower-quality housing or a less desirable neighborhood) that further diminish opportunities for health promotion and social connection, ultimately limiting both general health^52^ and physical activity.^53^ Although previous research^54^ has found an ecological association between neighborhood eviction rates and lower participation in leisure-time exercise, our study is the first, to our knowledge, to find an association between cost-driven moves and diminished physical activity at the individual level.

The associations of cost-driven moves with fewer preventive care visits and more ED visits are consistent with findings from a previous study reporting an association between cost-driven moves and unmet medical needs^23^ and implicate this facet of the affordable housing crisis in potentially having an important role in avoidable emergency health care use and spending.^55,56^ The fact that associations with these 2 health care use outcomes did not differ by move type suggests that material and geographic disruptions related to moving (cost-driven or otherwise) may be sufficient to make people delay establishing health care with a new clinician and/or deprioritize preventive and chronic health care amid competing needs.^23,57^

Overall, our results revealed an association between cost-driven moves and adverse health-related outcomes and behaviors of concern to clinicians, health care systems, public health officials, and community health advocates. Causal mechanisms should be explored in future research using longitudinal methods to characterize those who make cost-driven moves before and after their moves and to compare health-related outcomes between cost-burdened renters who subsequently do or do not move. Additional investigation is also warranted to consider how structural factors may be associated with disparate health outcomes across population subgroups and to explore strategies for prevention. Potential approaches to mitigate excessive housing costs include emergency rental assistance, longer-term rent subsidies, rent stabilization policies, affordable housing development incentives, social housing models, and revisions to zoning constraints on housing supply (especially if implemented in high-opportunity exclusionary neighborhoods and in conjunction with increased tenant protections).^58,59,60^ Laws to guarantee that renters with low income have access to lawyers and are protected against unjust evictions, landlord harassment, and source-of-income discrimination could also help prevent displacement.^59,61^ Such efforts likely need to be accompanied by policies to enhance income stability, such as through expanded access to unemployment insurance, the earned income tax credit,^62^ and the child tax credit.^63^ In addition, improved access to health care, such as through California’s recent expansion of Medicaid to middle-aged adults with low income regardless of immigration status,^64^ could help mitigate health care disruption and facilitate referrals to public benefits and community resources during and after the turbulence of a move.

Given the disparate prevalence of cost-driven moves by race, ethnicity, and other socioeconomic strata, which might result from differing economic opportunities and/or discrimination in housing, interventions to prevent cost-driven moves could help to interrupt a pathway by which structural racism produces health disparities. Combatting the upstream structural factors associated with economic inequality will be more challenging, but conversations around reparations offer a potential path forward.^65^

### Limitations

This study has several limitations. First, our primary exposure did not distinguish whether cost-driven moves were precipitated by changes in participants’ housing costs, income, or both. Second, due to potential ambiguity in survey item wording, it is also likely that our cost-driven moves variable may have missed some participants who were evicted due to nonpayment of rent, although sensitivity analyses examining the broader category of housing-related moves provided consistent results. Incorporating more specific measures of forced or reactive moves^15^ into future population health surveys could be instructive. Third, results may be biased toward the null because the CHIS excluded people without a telephone and individuals experiencing homelessness,^31^ who may be most vulnerable to harm from displacement. Fourth, we could not measure the characteristics of participants or their neighborhoods before the move occurred, nor could we exclude reverse causality; it is likely that in some cases, poor health led to cost-driven moves by restricting workforce participation and exacerbating health care debt.^66,67,68^ Fifth, although we controlled for many covariates, we could not confirm whether participants in the comparison groups had burdensome housing costs, and our analyses may be subject to residual confounding. Results should be interpreted as strictly associational. Further research is needed to explore the extent to which the observed associations may reflect causal effects specific to moving in the context of unaffordable housing costs, the broader consequences of experiencing unaffordable housing costs, the implications of poor health for economic precariousness and housing insecurity, or other factors associated with both cost-driven moves and health.

## Conclusions

In this cross-sectional study of California renters and other nonhomeowners, participants who recently moved due to unaffordable housing costs experienced worse outcomes among multiple domains of health compared with those who did not move and those who moved for non–cost-driven reasons. The widespread shortage of affordable housing thus confronts many renters with a daunting problem. Staying in unaffordable housing creates income constraints that may have negative consequences for health, but moving away from unaffordable housing is also associated with poor health-related outcomes. Policies to alleviate housing shortages, enhance housing affordability and stability, and improve access to health care and social services have the potential to improve population health equity by preventing or reducing the incidence of cost-driven moves.

## Notes

Supplement 1. **eTable 1.** Definitions of Census Tract Variables Assessed as Potential Moderators **eTable 2.** Weighted Prevalence of Reasons for Moving Among California Renters and Other Nonhomeowners Who Moved in the Past 3 Years **eTable 3.** Definitions of Control Variables Included in Multivariable Models **eTable 4.** Adjusted Associations Between Housing-Related Residential Moves and Health-Related Outcomes Relative to No Residential Move and Non–Housing-Related Residential Moves (Sensitivity Analyses) **eTable 5.** Adjusted Associations Between Cost-Driven Residential Moves and Alternate Specifications of Health-Related Outcomes Relative to No Residential Move (Sensitivity Analyses)
eReferences

Supplement 2.
Data Sharing Statement
