# Supplementary material for: Association of Cost-Driven Residential Moves With Health-Related Outcomes Among California Renters
Source: JAMA Netw Open. 2023 Mar 14;6(3):e232990. doi: 10.1001/jamanetworkopen.2023.2990 (PMC10015305; doi:10.1001/jamanetworkopen.2023.2990)
Supplement: Supplement 1. — eTable 1. Definitions of Census Tract Variables Assessed as Potential Moderators eTable 2. Weighted Prevalence of Reasons for Moving Among California Renters and Other Nonhomeowners Who Moved in the Past 3 Years eTable 3. Definitions of Control Variables Included in Multivariable Models eTable 4. Adjusted Associations Between Housing-Related Residential Moves and Health-Related Outcomes Relative to No Residential Move and Non–Housing-Related Residential Moves (Sensitivity Analyses) eTable 5. Adjusted Associations Between Cost-Driven Residential Moves and Alternate Specifications of Health-Related Outcomes Relative to No Residential Move (Sensitivity Analyses) eReferences [file jamanetwopen-e232990-s001.pdf]

Supplementary Online Content

Chen KL, Wisk LE, Nuckols TK, et al. Association of cost-driven residential moves with health-related outcomes among California renters. *JAMA Netw Open*. 2023;6(3):e232990. doi:10.1001/jamanetworkopen.2023.2990

eTable 1. Definitions of Census Tract Variables Assessed as Potential Moderators

eTable 2. Weighted Prevalence of Reasons for Moving Among California Renters and Other Nonhomeowners Who Moved in the Past 3 Years

eTable 3. Definitions of Control Variables Included in Multivariable Models

eTable 4. Adjusted Associations Between Housing-Related Residential Moves and Health-Related Outcomes Relative to No Residential Move and Non-Housing-Related Residential Moves (Sensitivity Analyses)

eTable 5. Adjusted Associations Between Cost-Driven Residential Moves and Alternate Specifications of Health-Related Outcomes Relative to No Residential Move (Sensitivity Analyses)

eReferences

This supplemental material has been provided by the authors to give readers additional information about their work.

© 2023 Chen KL et al. *JAMA Network Open*.

eTable 1. Definitions of Census Tract Variables Assessed as Potential Moderators

Census Tract Variable	Details
Social Vulnerability Index (SVI), quartiles	*4-level categorical variable*: California-wide quartiles of the Social Vulnerability Index (2016 version) developed by the Centers for Disease Control and Prevention (CDC)[1] to summarize social factors (socioeconomic status; household composition; race, ethnicity, and language; and housing and transportation) that confer community-level vulnerability or resilience to disasters.
Incarceration rate, quartiles	*4-level categorical variable*: California-wide quartiles of a measure (2020 version) developed for the Opportunity Atlas[2] to estimate the proportion of children born between 1978-1983 who grew up in a given census tract who were incarcerated in 2010.
Walkability, quartiles	*4-level categorical variable*: California-wide quartiles of the National Walkability Index (2013 version) developed by the Environmental Protection Agency[3] to summarize factors that make neighborhoods inviting to pedestrians.
Park access, quartiles	*4-level categorical variable*: California-wide quartiles of the National Environmental Public Health Tracking Network Access to Parks Indicator (2015 version) developed by the CDC[4,5] to estimate the proportion of census tract residents living within 0.5 miles of a park or other green space.
Housing and transportation affordability, quartiles	*4-level categorical variable*: California-wide quartiles of the Housing + Transportation Affordability Index (H+T Index, 2016 version) developed by the Center for Neighborhood Technology[6] to estimate location affordability relative to area median income. Based on the estimated costs of both housing and transportation, taking into account expected commute distances.

© 2023 Chen KL et al. *JAMA Network Open*.

eTable 2. Weighted Prevalence of Reasons for Moving Among California Renters and Other Nonhomeowners Who Moved in the Past 3 Years

Main reason for moving among recent movers (n=25,088)[a]	Column %[a]
Other housing related (n=6,635)	24.6
Couldn't afford mortgage/rent (n=3,747)	15.4
Work related (n=2,880)	13.0
Better neighborhood/less crime (n=2,506)	10.2
Change in marital/relationship status (n=1,700)	6.8
To establish own household (n=1,372)	5.6
To attend or leave college (n=962)	4.6
Changes in renting/lease or roommate issues (n=684)	2.4
Closer to family or family-related reasons (n=726)	2.0
For child's education (n=296)	1.1
Health or medical reasons (n=70)	0.2
Other (n=3,510)	14.2

[a]Note: All sample sizes displayed are unweighted, while all frequencies are weighted. Column may not add up to 100% due to rounding. Recent moves defined as moves in the past 3 years.

© 2023 Chen KL et al. *JAMA Network Open*.

eTable 3. Definitions of Control Variables Included in Multivariable Models

Control Variable	Type and Definition[a]
Gender	*Dichotomous*: female or male
Age	*Continuous:* years of age. We also included a term for age squared to account for possible non-linear relationships between age and the outcomes.
Race and ethnicity	*Categorical:* Asian non-Hispanic, Black non-Hispanic, Hispanic, White non-Hispanic or other non-Hispanic (including non-Hispanic American Indian or Alaska Native, non-Hispanic Native Hawaiian or other Pacific Islander, non-Hispanic participants describing their race as “other,” and non-Hispanic participants selecting two or more races).
Family composition	*Categorical:* single without children, married without children, single parent, or married parent.
Employment status	*Categorical:* Full-time, part-time, unemployed (looking for work), or unemployed (not looking for work)
Educational attainment	*Continuous*: years of education completed, estimated from categories reported by respondents, using midpoint of range if applicable (on formal education= 0, grade 1-8= 4.5, grade 9-11= 10, grade 12 or high-school diploma = 12, some college, vocational school, or AA or AS degree= 14, BA or BS degree= 16, some grad school or MA or MS degree= 18, PhD or equivalent= 20)
Income	*Continuous:* log of total household income as % of the federal poverty level (FPL) corresponding to each survey year
Citizenship	*Categorical:* US-born citizen, naturalized citizen, non-citizen with green card, or non-citizen, no green card
Limited English proficiency	*Dichotomous:* yes vs. no (defined as yes for participants who said that they speak English “not well” or “not at all”)
Housing type	*Dichotomous:* rent vs. other arrangement (sample excludes people who own their homes)
Urbanicity	*Dichotomous:* rural vs. urban neighborhood, assigned by CHIS using the Nielsen-Claritas urbanicity model according to respondent’s ZIP code
Health insurance type[b]	*Categorical:* Employer-sponsored insurance, Medicaid, Medicare or other public insurance alone, dual Medicaid and Medicare, Medicare plus other coverage, privately purchased insurance, or not insured

[a]Note: all variables were self-reported in the California Health Interview Survey except for urbanicity, which was assigned by the CHIS.

[b]Included in models of health services use (preventive visits and emergency department visits) only. The remaining control variables were included in all multivariable models.

© 2023 Chen KL et al. *JAMA Network Open*.

eTable 4. Adjusted Associations Between Housing-Related Residential Moves and Health-Related Outcomes Relative to No Residential Move and Non-Housing-Related Residential Moves (Sensitivity Analyses)[a]

	Comparison of adjusted health associations between **housing-related move vs. no move**		Comparison of adjusted health associations between **housing-related vs. non-housing-related moves**	
	Adjusted outcome for **no move** (ref.) n=27,558	Adjusted difference in outcome associated with **housing-related move** n=11,066	Adjusted outcome for **non-housing-related move** (ref.) n=14,022	Adjusted difference in outcome associated with **housing-related move** n=11,066
**Outcome**	*%*	*difference in %-points (95% CI)*	*%*	*difference in %-points (95% CI)*
General health is good, very good, or excellent	74.5	-0.4 (-1.9 to 1.2)	75.1	-1.0 (-2.7 to 0.8)
Psychological distress				
Severe	10.7	1.5[c] (0.7 to 2.3)	11.5	-1.8 (-3.8 to 0.2)
Moderate	30.8	2.1[c] (1.0 to 3.2)	31.9	1.0 (-0.1 to 2.2)
Low	58.5	-3.6[c] (-5.4 to -1.7)	56.7	0.8 (-0.1 to 1.6)
Preventive visit	68.1	-3.6[b] (-5.9 to -1.3)	65.9	-1.4 (-3.8 to 1.0)
ED visit	20.6	3.2[c] (1.6 to 4.8)	24.5	-0.8 (-2.6 to 1.0)
Walking for leisure, minutes[d]	96.7	-8.8 (-17.5 to 0.0)	93.1	-5.2 (-13.5 to 3.2)

Abbreviations: CI= confidence interval. ED= emergency department.
[a]Housing-related moves includes moves in the past 3 years attributed to: "couldn't afford rent," "changes in renting/lease or roommate issues," or "other housing-related" reasons. Non-housing-related moves includes moves in the past 3 years attributed to any other reason. "No move" indicates no move in the past 3 years. Unweighted sample sizes are shown; all other values represent weighted estimates. All models adjusted for gender, race and ethnicity, family composition, age, age squared, employment, educational attainment, income, housing type, urbanicity, limited English proficiency, citizenship, and survey year. Preventive visit and emergency department visit models were additionally adjusted for health insurance type.
[b]p<0.01
[c]p<0.001
[d]Outcomes reported as minutes (rather than %) and difference in minutes (95% CI) (rather than difference in %-points [95% CI]).

© 2023 Chen KL et al. *JAMA Network Open*.

eTable 5. Adjusted Associations Between Cost-Driven Residential Moves and Alternate Specifications of Health-Related Outcomes Relative to No Residential Move (Sensitivity Analyses)[a]

	Comparison of adjusted health associations between **cost-driven move vs. no move**		Comparison of adjusted health associations between **cost-driven vs. non-cost-driven moves**	
**Outcome**	Adjusted outcome for **no move** (ref.) n=27,558 *%*	Adjusted difference in outcome associated with **cost-driven move** n=3,747 *difference in %-points (95% CI)*	Adjusted outcome for **non-cost-driven move** (ref.) n=21,341 *%*	Adjusted difference in outcome associated with **cost-driven move** n=3,747 *difference in %-points (95% CI)*
General health[b]				
Poor	5.4	1.0[h] (0.4 to 1.7)	5.2	1.2[h] (0.6 to 1.8)
Fair	20.1	2.3[h] (1.0 to 3.7)	19.6	2.8[h] (1.5 to 4.2)
Good	32.8	0.9[h] (0.5 to 1.3)	32.6	1.1[h] (0.7 to 1.6)
Very good	26.5	-1.9[h] (-3.0 to -0.8)	26.9	-2.3[h] (-3.4 to -1.2)
Excellent	15.3	-2.4[h] (-3.7 to -1.1)	15.8	-3.0[h] (-4.2 to -1.7)
Psychological distress, K6 score[c]	4.2	0.6[h] (0.4 to 0.9)	4.4	0.5[h] (0.2 to 0.8)
Preventive visit (past 2 years)[d]	80.8	-3.0 (-6.1 to 0.1)	79.3	-1.5 (-4.6 to 1.6)
Number of ED visits[e]	0.4	0.2[g] (0.1 to 0.3)	0.5	0.1 (-0.0 to 0.2)
Regularly walked for leisure[f]	11.7	-1.6 (-3.5 to 0.2)	10.8	-0.7 (-2.6 to 1.1)

Abbreviations: CI= confidence interval. ED= emergency department.

[a]Unweighted sample sizes are shown; all other values represent weighted estimates. The inflation equation for the zero-inflated negative binomial model for emergency department visits adjusted for move status, gender, race and ethnicity, age, employment, education, income, urbanicity, limited English proficiency, citizenship, and health insurance. All other models were adjusted for gender, race, family composition, age, age squared, employment, educational attainment, log of income as percent of federal poverty level, housing type, urbanicity, limited English proficiency, citizenship, and survey year. Preventive visit and emergency department visit models were additionally adjusted for health insurance type.

[b]General health modeled as 5-level ordinal variable using partial proportional odds models.[7]

[c]Kessler-6 (K6) score for psychological distress modeled as continuous outcome using ordinal linear regression. Outcomes reported as K6 points (rather than %) and difference in K6 points (95% CI) (rather than difference in %-points [95% CI]).

[d]Proportion with any preventive visit in the past 2 years modeled using logistic regression.

[e]Number of emergency department visits in the past 12 months modeled as count variable using zero-inflated negative binomial models. Outcomes reported as count of ED visits (rather than %) and difference in count of ED visits (95% CI) (rather than difference in %-points [95% CI]).

[f]Proportion that regularly walked for leisure (defined as having walked for leisure for ≥30 minutes ≥5 times in the last 7 days) modeled using logistic regression.

[g]$p<0.01$

[h]$p<0.001$

© 2023 Chen KL et al. *JAMA Network Open*.

eReferences

1. Centers for Disease Control and Prevention/ Agency for Toxic Substances and Disease Registry/ Geospatial Research, Analysis, and Services Program. CDC/ATSDR Social Vulnerability Index 2016 Database, California. Published online February 13, 2020.

2. Chetty R, Friedman J, Hendren N, Jones M, Porter S. *The Opportunity Atlas: Mapping the Childhood Roots of Social Mobility*. National Bureau of Economic Research; 2018:w25147. doi:10.3386/w25147

3. Thomas J, Zeller L. *National Walkability Index: Methodology and User Guide*. U.S. Environmental Protection Agency; 2021. Accessed November 16, 2021. https://www.epa.gov/sites/default/files/2021-06/documents/national_walkability_index_methodology_and_user_guide_june2021.pdf

4. Ussery EN, Yngve L, Merriam D, et al. The National Environmental Public Health Tracking Network Access to Parks Indicator: A National County-Level Measure of Park Proximity. *J Park Recreat Admi*. 2016;34(3):52-63. Accessed January 31, 2022. https://www.ncbi.nlm.nih.gov/pmc/articles/PMC5580831/

5. Centers for Disease Control and Prevention. Indicator: Access to Parks and Public Elementary Schools. National Environmental Public Health Tracking. Published May 31, 2022. Accessed January 31, 2022. https://ephtracking.cdc.gov/indicatorPages?selectedContentAreaAbbreviation=14&selectedIndicatorId=80&selectedMeasureId=

6. Center for Neighborhood Technology. H + T Index Methods. Published online 2017.

7. Williams R. Generalized Ordered Logit/Partial Proportional Odds Models for Ordinal Dependent Variables. *The Stata Journal*. 2006;6(1):58-82. doi:10.1177/1536867X0600600104

© 2023 Chen KL et al. *JAMA Network Open*.
