# Supplementary material for: Association of Cost-Driven Residential Moves With Health-Related Outcomes Among California Renters
Source: JAMA Netw Open. 2023 Mar 14;6(3):e232990. doi: 10.1001/jamanetworkopen.2023.2990 (PMC10015305; doi:10.1001/jamanetworkopen.2023.2990)
Supplement: Supplement 2. — Data Sharing Statement [file jamanetwopen-e232990-s002.pdf]

Data Sharing Statement

Chen. Association of Cost-Driven Residential Moves With Health-Related Outcomes Among California Renters. *JAMA Netw Open*. Published March 14, 2023. doi:10.1001/jamanetworkopen.2023.2990

Data
Data available: No

Additional Information
Explanation for why data not available: Data are managed by the California Health Interview Survey (CHIS). We analyzed the restricted-use CHIS dataset that are only available by application to the CHIS Data Access Center.
